# Environmental exposure of heavy metal (lead and cadmium) and hearing loss: data from the Korea National Health and Nutrition Examination Survey (KNHANES 2010–2013)

**DOI:** 10.1186/s40557-018-0237-9

**Published:** 2018-04-17

**Authors:** Gu Hyeok Kang, Jun Young Uhm, Young Gon Choi, Eun Kye Kang, Soo Young Kim, Won Oh Choo, Seong Sil Chang

**Affiliations:** Department of Occupational & Environmental Medicine, Eulji University Hospital, 95 Dunsanseo-ro, Seo-gu, Daejeon, 35233 Republic of Korea

**Keywords:** Hearing loss, Heavy metals, Lead, Cadmium, KNHANES

## Abstract

**Background:**

Lead and cadmium have been identified as risk factors for hearing loss in animal studies, but large-scale studies targeting the general human population are rare. This study was conducted to investigate the link between heavy metal concentrations in blood and hearing impairment, using a national population-based survey.

**Methods:**

The study participants comprised 6409 Koreans aged 20 or older, who were included in the Fifth and Sixth Korea National Health and Nutrition Examination Surveys (KNHANES 2010–2013). Hearing impairment was categorized into two types, low- and high-frequency hearing impairment, using pure tone audiometry. Low-frequency hearing impairment was defined as having a binaural average of hearing thresholds for 0.5, 1, and 2 kHz exceeding 25 dB, and high-frequency hearing impairment was defined as having a binaural average of hearing thresholds for 3, 4, and 6 kHz exceeding 25 dB. The blood levels of heavy metals (lead and cadmium) were classified into quartiles. Cross-sectional association between hearing impairment and the level of heavy metals (lead and cadmium) was examined in both sexes. Multivariate logistic regression was used to obtain adjusted odds ratios (ORs) and 95% confidence intervals (CIs).

**Results:**

Among men, the prevalence of low- and high- frequency hearing impairment was 13.9% and 46.7%, respectively, which was higher than the prevalence among women (11.8% and 27.0%, respectively). Regarding lead, the adjusted OR of high-frequency hearing impairment for the highest blood level group versus the lowest group was significant in both men (OR = 1.629, 95% CI = 1.161–2.287) and women (OR = 1.502, 95% CI = 1.027–2.196), after adjusting for age, body mass index, education, smoking, alcohol consumption, exercise, diagnosis of diabetes mellitus, hypertension, and noise exposure (occupational, loud, firearm noises). No links were found between blood lead levels and low-frequency hearing impairment, or between blood cadmium levels and low- or high-frequency hearing impairment in either sex.

**Conclusions:**

The present study findings suggest that even exposure to low-level lead is a risk factor for high-frequency hearing loss. A prospective epidemiologic study should be conducted to identify the causal relationship between human health and exposure to heavy metals, and efforts to reduce heavy metal exposure in the general population should continue.

## Background

Hearing impairment is one of the most common health conditions, and is increasing in aging societies [1, 2]. According to the World Health Organization (WHO), 360 million people worldwide and approximately one third of the older population (65 years or older) suffer from hearing impairment [3]. In a study based on the 2010–2012 Korea National Health and Nutrition Examination Surveys (KNHANES), it was estimated that people with mild hearing impairment comprise 20.5% of the population over the age of 19 years, and 70% of the population over the age of 65 years [4]. Hearing impairment can cause social isolation due to communication problems in everyday life [5, 6], and is a serious public health issue in Korea.

Hearing impairment tends to increase rapidly with age [7]. Aside from aging, exposure to loud noise is a risk factor for hearing loss [8], and ototoxic chemicals also affect hearing ability [9–11]. Numerous studies have reported the effect of heavy metals on hearing loss. Jones et al. argued that lead is an ototoxic heavy metal and that lead exposure results in degeneration of the inner ear receptor cells and decreases the conduction function of auditory nerve cells [12]. Cadmium is reported to cause apoptosis of inner ear receptor cells and alter their arrangement, resulting in an increased hearing threshold [13, 14]. Prasher suggested multiple ototoxic effects of lead and cadmium [15]. Additionally, a limited number of epidemiologic studies have been conducted. In a study on the general population, Park et al. confirmed a link between low-level lead exposure and hearing impairment [16]. The relationship between hearing impairment and exposure to lead and cadmium was confirmed in a study by Shargorodsky et al. of American adolescents [17], and in a study of American adults by Choi et al. [18].

Heavy metals, such as lead and cadmium, are present not only in factory-manufactured products, but also in the environment within air, water, food, soil, and dust [19–21]. Industrialization has surreptitiously exposed human bodies to heavy metal [22]. Once heavy metal is absorbed into the body, it is stored in the tissues, because the human body has a limited capacity to effectively eliminate it, due to a half-life lasting several decades [23]. Consequently, the link between hearing impairment and heavy metal levels requires close examination. Currently, lead and cadmium concentration levels in Korea continue to decrease but remain higher when compared to developed countries such as the United States (US) and Canada [24]. Accordingly, the link between heavy metals and hearing impairment should be carefully monitored in Korea.

Several studies have examined the relationship between hearing impairment and heavy metals. However, the number of studies on the general population is limited and the number of epidemiologic studies conducted within the Korean population is even fewer. Accordingly, the present study was conducted to investigate the link between exposure to lead and cadmium and hearing impairment using KNHANES data.

## Methods

### Participants

Data from the fifth and sixth KNHANES, gathered from 2010 to 2013, was used for the analyses. The KNHANES is a multistage stratified complex design survey of a representative sample of the entire Korean population, conducted by the Korea Centers for Disease Control and Prevention. Trained interviewers and laboratory technicians conducted surveys in households, including administering questionnaires, performing health examinations, and collecting blood samples. The section for health behaviors, such as smoking and alcohol consumption, was self-administered. The total number of respondents was 33,552.

Of those, 6582 participants aged 20 years or older underwent pure tone audiometry and blood tests to assess the level of heavy metals (lead and cadmium). After excluding those with missing data, a total of 6409 participants were selected as study participants.

### Hearing impairment

To examine the respondents hearing condition, pure tone audiometry was performed. Both ears were tested at 0.5, 1, 2, 3, 4, and 6 kHz, in a soundproof booth, using the Entomed SA 203. WHO defines hearing as normal if, in the better hearing ear, the average of hearing thresholds at 0.5, 1, 2, and 4 kHz is under 25 dB [25]. Until recently, this definition has been widely used as the criterion to determine an individual’s hearing condition in everyday life. However, potential hearing impairment of one ear may be neglected when the hearing threshold is under 25 dB in the other ear. To account for this, some studies have defined hearing impairment by taking the binaural average of hearing thresholds in preference to relying on the hearing ability of one ear only [26], while another study measured 2 types of hearing impairment, low- and high-frequency hearing impairment [27]. Based on information from these studies, we used the binaural pure-tone average threshold and computed two binaural averages, one across 0.5, 1, and 2 kHz and the other across 3, 4, and 6 kHz to determine the low- and high-frequency thresholds. Hearing impairment was then determined according to whether an average threshold exceeded 25 dB in the respective frequency band.

### Measurement of lead and cadmium in whole blood

To measure heavy metal blood levels, blood samples were collected into standard commercial evacuated tubes coated with sodium heparin (Vacutainer). Blood lead and cadmium levels were measured via graphite furnace atomic absorption spectrometry (Perkin Elmer AAnalyst 600, Turku, Finland). For internal quality assurance, the analytical equipment was controlled with respect to the standard reference material from Whole Blood Metals Control (BIO-RAD, USA). The blood levels of each heavy metal were classified into quartiles separately for men and women, and geometric means with standard error were computed according to the quartile rank.

### Other variables

In the analysis, we included factors suggested by previous studies as affecting hearing impairment. Some studies suggested that cerebro-cardiovascular risk factors, such as obesity and smoking, have a negative effect on hearing ability [28, 29], because the cochlea is vulnerable to ischemic changes [30]. On the other hand, moderate exercise and moderate alcohol consumption are reported to have a beneficial effect on hearing ability [31, 32].

Participants’ ages were grouped in 10-year intervals, starting from age 20, and participants aged 70 or higher were collectively gathered as one group. Body mass index (BMI) under 18.5 kg/m^2^ was classified as underweight, BMI between 18.5 and 25 kg/m^2^ as normal weight, and BMI over 25 kg/m^2^ as overweight. The education level was categorized as less than high school education; high school graduation; and more than high school education. Cigarette smoking was classified as follows: a non-smoker was defined as never having smoked more than 100 cigarettes; an ex-smoker was defined as having smoked 100 or more cigarettes but currently not smoking; and a current smoker was defined as having smoked 100 or more cigarettes and still currently smoking. Alcohol consumption was defined as: a non-drinker having never consumed alcohol, a light drinker as consuming alcohol less than twice per week or having fewer than 7 glasses (5 glasses for women) when drinking, and a heavy drinker as drinking more than twice per week or having 7 or more glasses (5 glasses for women) when drinking. Participants who performed intense physical activities 3 days or more per week and, with exercise, felt the effects of bodily exertions more than usual, or who performed intense exercises requiring heavy breathing for 30 min or more, were classified into an exercise group, and all others classified into a non-exercise group. Regarding noise exposure, participants who had been exposed to noise at their workplace over a 3-month period or exposed to loud noises such as car horns, machinery, and loud music at places other than the workplace for 5 h or more per week were classified into a noise exposure group. Those who had been exposed to firearm noise were also classified into the noise exposure group. Finally, regarding diabetes and hypertension, participants were categorized into one of two groups, respectively, depending on whether they had been diagnosed with diabetes or hypertension by a physician.

### Statistical analyses

Weighted complex sampling analysis available from SPSS v.18.0 was used for data analysis. Regarding the general characteristics of participants, the mean and sample size for each variable was examined by sex. A chi-square test was performed to examine the distributions and t-tests performed to compare means. The relationship between each independent variable and hearing impairment was examined using a chi-square test. This test was conducted separately for low- and high-frequency hearing impairment and by sex. To examine sex-specific relationships between low- and high-frequency hearing impairment and heavy metal levels in blood, odds ratios (OR) were computed using complex sampling logistic regression. Statistical significance was defined as *p* < 0.05. In the first model, age, BMI, education, smoking, alcohol consumption, and exercise were included as adjusting variables, and in the second model, diabetes mellitus, and hypertension were additionally included as adjusting variables. The ORs in the final model were adjusted with exposures to occupational, loud, and firearm noises.

## Results

### Participants’ general characteristics

Participants totaled 6409, of whom 3185 (49.6%) were men and 3224 (50.4%) were women. The mean age across all participants was 47; the mean age for men was 46, and for women, 48. Table 1 shows the general characteristics of the study participants. Regarding factors in respect of lifestyle habits, the proportions of ex-smokers and current smokers were both high in men, as well as the proportion of heavy drinkers. Regarding education levels, the proportions of those who had graduated from high school and those who had received more than high school education were significantly higher among men than women (*p* < 0.001). Additionally, the proportions of those exposed to occupational, loud, and firearm noises were significantly higher in men than in women.

**Table 1:** General characteristics of the study population

Variables	Total n^a^	Male	Female	*p*-value^b^
Total	6409	3185	3224	
Age (years)				
Mean ± SE	47.1 ± 0.3	46.3 ± 0.3	48.0 ± 0.3	< 0.001
20–29	1025	493	532	0.002
30–39	1081	537	544	
40–49	1415	709	706	
50–59	1454	717	737	
60–69	1232	615	617	
70–87	202	114	88	
BMI (kg/m^2^)				
Mean ± SE	23.8 ± 0.1	24.2 ± 0.1	23.5 ± 0.1	< 0.001
Underweight	246	68	178	< 0.001
Normal	4071	1936	2135	
Obese	2092	1181	911	
Education				
≤ Middle school	1861	751	1110	< 0.001
High school	2365	1226	1139	
≥ College	2183	1208	975	
Smoking				
Non-smoker	3650	723	2927	< 0.001
Ex-smoker	1176	1063	113	
Current smoker	1583	1399	184	
Alcohol				
None	638	104	534	< 0.001
Light drinker	4128	1923	2205	
Heavy drinker	1643	1158	485	
Exercise				
No	4771	2271	2500	< 0.001
Yes	1638	914	724	
Current diagnosis of diabetes mellitus				
No	5980	2936	3044	0.069
Yes	429	249	180	
Current diagnosis of hypertension				
No	5237	2573	2664	0.202
Yes	1172	612	560	
Occupational noise exposure				
No	5496	2538	2958	< 0.001
Yes	913	647	266	
Loud noise exposure				
No	6267	3095	3172	0.008
Yes	142	90	52	
Firearm noise exposure				
No	4750	1642	3108	< 0.001
Yes	1659	1543	116	

SE standard error

^a^unweighted count, ^b^tested by chi-square test

### The prevalence of low- and high-frequency hearing impairment

Tables 2 and 3 show the male and female prevalence of low- and high-frequency hearing impairment (defined, respectively, as a binaural average of hearing thresholds for 0.5, 1 and 2 kHz, and for 3, 4, and 6 kHz exceeding 25 dB), broken down for each of the variables. The prevalence of low- and high-frequency hearing impairment in men was 13.9% and 46.7%, respectively, which was higher than the prevalence in women (11.8% and 27.0%, respectively).

**Table 2:** Characteristics by hearing impairment in male subjects

Variables	Low frequency hearing impairment				High frequency hearing impairment			
	Normal^a^	Impaired^a^	Rate^b^	p-value^c^	Normal^a^	Impaired^a^	Rate^b^	*p* -value^c^
Total	2743	442	13.9		1697	1488	46.7	
Age								
20–29	486	7	1.4	< 0.001	466	27	5.5	< 0.001
30–39	525	12	2.2		455	82	15.3	
40–49	680	29	4.1		444	265	37.4	
50–59	601	116	16.2		244	473	66	
60–69	406	209	34		84	531	86.3	
70–87	45	69	60.5		4	110	96.5	
BMI (kg/m^2^)								
Underweight	56	12	17.6	0.086	39	29	42.6	0.610
Normal	1650	286	14.8		1020	916	47.3	
Obese	1037	144	12.2		638	543	46	
Education								
≤ Middle school	514	237	31.6	< 0.001	159	592	78.8	< 0.001
High school	1086	140	11.4		691	535	43.6	
≥ College	1143	65	5.4		847	361	29.9	
Smoking								
Non-smoker	664	59	8.2	< 0.001	483	240	33.2	< 0.001
Ex-smoker	860	203	19.1		426	637	59.9	
Current smoker	1219	180	12.9		788	611	43.7	
Alcohol								
None	77	27	26	< 0.001	42	62	59.6	< 0.001
Light drinker	1613	310	16.1		914	1009	52.5	
Heavy drinker	1053	105	9.1		741	417	36	
Exercise								
No	1941	330	14.5	0.093	1195	1076	47.4	0.239
Yes	802	112	12.3		502	412	45.1	
Current diagnosis of diabetes mellitus								
No	2558	378	12.9	< 0.001	1639	1297	44.2	< 0.001
Yes	185	64	25.7		58	191	76.7	
Current diagnosis of hypertension								
No	2289	284	11	< 0.001	1528	1045	40.6	< 0.001
Yes	454	158	25.8		169	443	72.4	
Occupational noise exposure								
no	2222	316	12.5	< 0.001	1413	1125	44.3	< 0.001
yes	521	126	19.5		284	363	56.1	
Loud noise exposure								
no	2667	428	13.8	0.640	1645	1450	46.8	0.386
yes	76	14	15.6		52	38	42.2	
Firearm noise exposure								
no	1425	217	13.2	0.265	884	758	46.2	0.517
yes	1318	225	14.6		813	730	47.3	

^a^unweighted count

^b^prevalence rate

^c^tested by chi-square test

**Table 3:** Characteristics by hearing impairment in female subjects

Variables	Low frequency hearing impairment				High frequency hearing impairment			
	Normal^a^	Impaired^a^	Rate^b^	*p*-value^c^	Normal^a^	Impaired^a^	Rate^b^	*p*-value^c^
Total	2845	379	11.8		2354	870	27.0	
Age								
20–29	525	7	1.3	< 0.001	519	13	2.4	< 0.001
30–39	535	9	1.7		522	22	4	
40–49	676	30	4.2		614	92	13	
50–59	641	96	13		479	258	35	
60–69	421	196	31.8		209	408	66.1	
70–87	47	41	46.6		11	77	87.5	
BMI (kg/m^2^)								
Underweight	167	11	6.2	< 0.001	156	22	12.4	< 0.001
Normal	1906	229	10.7		1603	532	24.9	
Obese	772	139	15.3		595	316	34.7	
Education								
≤ Middle school	836	274	24.7	0.031	510	600	54.1	< 0.001
High school	1058	81	7.1		938	201	17.6	
≥ College	951	24	2.5		906	69	7.1	
Smoking								
Non-smoker	2569	358	12.2	0.062	2114	813	27.8	0.004
Ex-smoker	105	8	7.1		88	25	22.1	
Current smoker	171	13	7.1		152	32	17.4	
Alcohol								
None	412	122	22.8	< 0.001	281	253	47.4	< 0.001
Light drinker	1962	243	11		1646	559	25.4	
Heavy drinker	471	14	2.9		427	58	12	
Exercise								
No	2185	315	12.6	0.006	1794	706	28.2	0.003
Yes	660	64	8.8		560	164	22.7	
Current diagnosis of diabetes mellitus								
No	2717	327	10.7	< 0.001	2272	772	25.4	< 0.001
Yes	128	52	28.9		82	98	54.4	
Current diagnosis of hypertension								
No	2444	220	8.3	< 0.001	2112	552	20.7	< 0.001
Yes	401	159	28.4		242	318	56.8	
Occupational noise exposure								
No	2610	348	11.8	0.957	2174	784	26.5	0.043
Yes	235	31	11.7		180	86	32.3	
Loud noise exposure								
No	2797	375	11.8	0.359	2318	854	26.9	0.535
Yes	48	4	7.7		36	16	30.8	
Firearm noise exposure								
no	2747	361	11.6	0.200	2288	820	26.4	< 0.001
yes	98	18	15.5		66	50	43.1	

^a^unweighted count

^b^prevalence rate

^c^tested by chi-square test

In men, the proportions of low- and high-frequency hearing impairment increased with age (low frequency: *p* < 0.001, high frequency: p < 0.001). Focusing on BMI and exercise habits, the prevalence of low-frequency hearing impairment had a lower tendency but not significantly lower in the obese group (*p* = 0.086) and in the exercise group (*p* = 0.093). Regarding education level, the prevalence of both low- and high-frequency hearing impairment decreased as the education level advanced (*p* < 0.001). Low- and high-frequency hearing impairment was less prevalent in the non-smoker group and in the heavy drinker group, as well as in the groups not diagnosed with diabetes and hypertension. In contrast, both low- and high-frequency hearing impairment was significantly more prevalent in the group exposed to occupational noise (*p* < 0.001). There was no significant difference in the groups exposed to loud and firearm noises.

In women, the proportions of low- and high-frequency hearing impairment increased with age (low frequency: *p* < 0.001, high frequency: p < 0.001). The prevalence of both low- and high-frequency impairment was significantly lower where BMI was lower and the education level was higher, as well as in the heavy drinker group and the groups not diagnosed with diabetes and hypertension. Exposure to loud noise did not show a significant difference, but the group exposed to occupational and firearm noise showed a significantly higher prevalence of high-frequency hearing impairment (*p* = 0.043, *p* < 0.001).

**Fig. 1:**
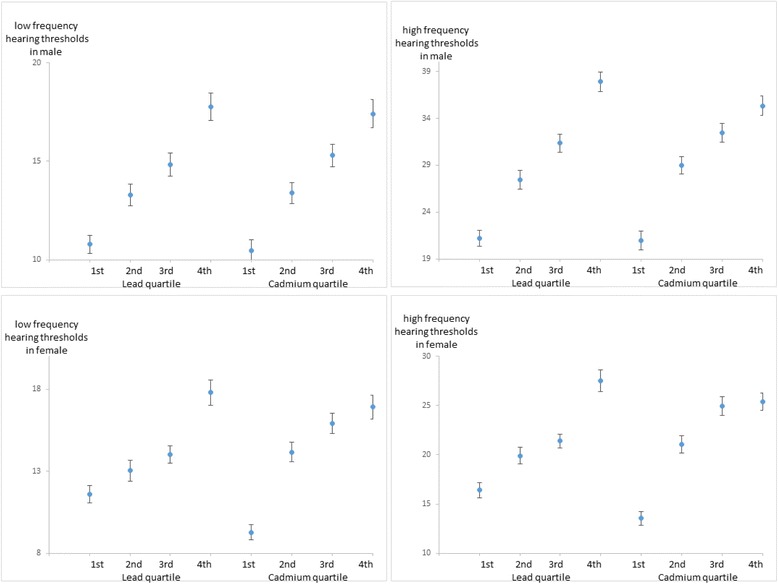
Binaural average of frequency hearing thresholds by quartile of lead, cadmium. Mean ± SD

Figure 1 shows the binaural average of frequency hearing thresholds of quartile groups based on blood lead level and blood cadmium level in male and female participants (mean ± SD). The figure displays that the hearing thresholds increase as the quartile group is higher in general.

### ORs of hearing impairment according to blood lead and cadmium levels

Tables 4 and 5 show the results of multivariate logistic regression analysis conducted for male and female participants. The tables display the ORs of low- and high-frequency hearing impairment for two sets of quartile groups, based on blood lead level and blood cadmium level, respectively.

**Table 4:** Adjusted ORs and 95% CI of hearing impairment by quartile of lead, cadmium in male

Analyte	<25th	25th to <50th	50th to <75th	≥75th
Lead				
Conc, μg/dL^a^	1.56 ± 0.01	2.22 ± 0.01	2.82 ± 0.01	4.22 ± 0.08
Case/n (low frequency)	80/796	96/797	111/796	155/796
Hearing thresholds(dB)^b^	10.8 ± 0.47	13.3 ± 0.56	14.8 ± 0.59	17.8 ± 0.70
Prevalence(%)^c^	9.2%	12.7%	16.2%	21.7%
Adjusted OR(95% CI)^d^	Referent	1.153 (0.761–1.747)	1.002 (0.644–1.559)	1.049 (0.694–1.585)
Adjusted OR(95% CI)^e^	Referent	1.15 (0.758–1.745)	0.998 (0.640–1.555)	1.045 (0.690–1.582)
Adjusted OR(95% CI)^f^	Referent	1.17 (0.772–1.772)	1.028 (0.661–1.597)	1.026 (0.677–1.556)
Case/n (high frequency)	242/796	322/797	404/796	520/796
Hearing thresholds(dB)^b^	21.2 ± 0.86	27.4 ± 0.99	31.4 ± 0.99	37.9 ± 1.05
Prevalence(%)^c^	28.6%	41.6%	51.3%	63.9%
Adjusted OR(95% CI)^d^	Referent	1.342 (0.988–1.823)	1.357 (0.972–1.894)	1.598 (1.14–2.238)
Adjusted OR(95% CI)^e^	Referent	1.352 (0.994–1.837)	1.365 (0.978–1.906)	1.614 (1.151–2.263)
Adjusted OR(95% CI)^f^	Referent	1.368 (1.006–1.859)	1.402 (1.005–1.955)	1.629 (1.161–2.287)
Cadmium				
Conc, μg/dL^a^	0.47 ± 0.01	0.79 ± 0.01	1.13 ± 0.01	1.88 ± 0.03
Case/n (low frequency)	57/797	99/797	130/798	156/793
Hearing thresholds(dB)^b^	10.5 ± 0.55	13.4 ± 0.53	15.3 ± 0.57	17.4 ± 0.71
Prevalence(%)^c^	8.4%	12.9%	17.1%	21.1%
Adjusted OR(95% CI)^d^	Referent	0.857 (0.532–1.379)	0.845 (0.54–1.322)	0.924 (0.567–1.505)
Adjusted OR(95% CI)^e^	Referent	0.855 (0.531–1.376)	0.84 (0.537–1.313)	0.922 (0.566–1.503)
Adjusted OR(95% CI)^f^	Referent	0.842 (0.524–1.354)	0.83 (0.533–1.291)	0.905 (0.556–1.473)
Case/n (high frequency)	221/797	362/797	433/798	472/793
Hearing thresholds(dB)^b^	20.9 ± 1.00	29.0 ± 0.94	32.5 ± 1.02	35.3 ± 1.04
Prevalence(%)^c^	27.4%	46.3%	52.3%	59.2%
Adjusted OR(95% CI)^d^	Referent	1.238 (0.895–1.712)	1.159 (0.828–1.623)	1.284 (0.892–1.848)
Adjusted OR(95% CI)^e^	Referent	1.236 (0.894–1.71)	1.163 (0.83–1.629)	1.283 (0.891–1.848)
Adjusted OR(95% CI)^f^	Referent	1.235 (0.891–1.711)	1.168 (0.833–1.638)	1.292 (0.896–1.865)

^a^Conc, weighted mean ± SE

^b^Binaural average of hearing thresholds, weighted mean ± SE

^c^Weighted percentages

^d^Adjusted for age, BMI, education, smoking, alcohol consumption, and exercise,

^e^Additional adjustment for diabetes mellitus, hypertension

^f^Additional adjustment for noise exposure (Occupational, loud, Firearm noise)

**Table 5:** Adjusted ORs and 95% CI of hearing impairment by quartile of lead, cadmium in female

Analyte	<25th	25th to <50th	50th to <75th	≥75th
Lead				
Conc, μg/dL^a^	1.12 ± 0.01	1.61 ± 0.01	2.11 ± 0.01	3.03 ± 0.03
Case/n (low frequency)	63//808	82/804	101/806	133/806
Hearing thresholds(dB)^b^	11.6 ± 0.53	13.0 ± 0.63	14.0 ± 0.52	17.8 ± 0.79
Prevalence(%)^c^	10.9%	11.1%	12.6%	20.6%
Adjusted OR(95% CI)^d^	Referent	1.312 (0.755–2.279)	1.302 (0.791–2.143)	0.957 (0.563–1.626)
Adjusted OR(95% CI)^e^	Referent	1.288 (0.74–2.24)	1.281 (0.773–2.123)	0.93 (0.544–1.589)
Adjusted OR(95% CI)^f^	Referent	1.271 (0.726–2.223)	1.308 (0.784–2.183)	0.932 (0.541–1.604)
Case/n (high frequency)	135/808	178/804	234/806	323/806
Hearing thresholds(dB)^b^	16.4 ± 0.75	19.9 ± 0.87	21.4 ± 0.70	27.5 ± 1.10
Prevalence(%)^c^	20.3%	24.2%	29.2%	44.7%
Adjusted OR(95% CI)^d^	Referent	0.937 (0.599–1.464)	1.009 (0.695–1.464)	1.488 (1.02–2.172)
Adjusted OR(95% CI)^e^	Referent	0.941 (0.602–1.471)	1.012 (0.698–1.467)	1.499 (1.028–2.187)
Adjusted OR(95% CI)^f^	Referent	0.947 (0.606–1.477)	1.013 (0.698–1.471)	1.502 (1.027–2.196)
Cadmium				
Conc, μg/dL^a^	0.57 ± 0.01	0.96 ± 0.01	1.36 ± 0.01	2.17 ± 0.03
Case/n (low frequency)	42/806	89/807	110/806	138/805
Hearing thresholds(dB)^b^	9.3 ± 0.45	14.2 ± 0.59	15.9 ± 0.63	16.9 ± 0.73
Prevalence(%)^c^	6.2%	13.4%	17%	18.5%
Adjusted OR(95% CI)^d^	Referent	0.873 (0.486–1.567)	0.916 (0.523–1.603)	0.768 (0.432–1.364)
Adjusted OR(95% CI)^e^	Referent	0.88 (0.489–1.586)	0.92 (0.524–1.616)	0.758 (0.426–1.349)
Adjusted OR(95% CI)^f^	Referent	0.875 (0.488–1.57)	0.913 (0.521–1.601)	0.757 (0.427–1.343)
Case/n (high frequency)	85/806	212/807	280/806	293/805
Hearing thresholds(dB)^b^	13.5 ± 0.69	21.1 ± 0.88	25.0 ± 0.94	25.4 ± 0.90
Prevalence(%)^c^	13.3%	28.4%	37.6%	38.6%
Adjusted OR(95% CI)^d^	Referent	1.225 (0.799–1.878)	1.302 (0.821–2.067)	1.425 (0.91–2.231)
Adjusted OR(95% CI)^e^	Referent	1.229 (0.799–1.89)	1.308 (0.821–2.082)	1.427 (0.908–2.244)
Adjusted OR(95% CI)^f^	Referent	1.248 (0.812–1.919)	1.325 (0.831–2.115)	1.426 (0.906–2.244)

^a^Conc, weighted mean ± SE

^b^Binaural average of hearing thresholds, weighted mean ± SE

^c^Weighted percentages

^d^Adjusted for age, BMI, education, smoking, alcohol consumption, and exercise,

^e^Additional adjustment for diabetes mellitus, hypertension

^f^Additional adjustment for noise exposure (Occupational, loud, Firearm noise)

In men, the ORs of low-frequency hearing impairment were not significant for the quartile groups based on blood lead levels, with the lowest group as a reference. However, the OR of high-frequency hearing impairment for the highest quartile group compared to the lowest group was significantly high, that is, 1.598 (95% confidence interval [CI] = 1.140–2.238), after adjusting for age, BMI, education, smoking, alcohol consumption, and exercise. The outcome was the same when diabetes mellitus and hypertension were added as adjusted variables. After adjusting for exposure to occupational, loud, and firearm noises, the ORs significantly increased for all groups, compared to the lowest blood lead group as a reference. Regarding blood cadmium levels, the OR of low- or high-frequency hearing impairment was not significant for any quartile groups, with the lowest group as a reference.

As with the men, the ORs of low-frequency hearing impairment were not significant in women for any quartile groups based on blood lead levels, with the lowest group as a reference. However, the OR of high-frequency hearing impairment for the highest quartile group with the lowest as a reference was significant, that is, 1.488 (95% CI = 1.02–2.172), when adjusted for age, BMI, education, smoking, alcohol consumption, and exercise. The result was the same when diabetes mellitus, hypertension, and exposures to occupational, loud, and firearm noises were additionally included as adjusted variables. In addition, in line with findings for the men, women’s ORs for any quartile groups, based on blood cadmium level with the lowest group as a reference, were not significant in either low- or high-frequency hearing impairment.

## Discussion

The objective of the present study was to investigate the link between heavy metals to which the general population is generally exposed and hearing impairment. In the study, there was no difference between the sexes in respect of any link between heavy metals and hearing impairment. Regarding the relationship between blood lead level and high-frequency hearing impairment, the risk of hearing impairment significantly increased in both sexes. However, with respect to low-frequency hearing impairment, significant results were not obtained. In addition, blood cadmium level did not show a significant result in either low- or high-frequency hearing impairment in either sex.

Several previous studies have reported associations between lead and cadmium levels, and hearing impairment. In a study within the general US population, Choi et al. found that the hearing threshold of the highest quintile groups, based on blood lead and cadmium levels, was higher compared to the respective lowest group, by approximately 18.6% (95% CI = 7.4%–31.1%) and 13.8% (95% CI = 4.6%–23.8%), respectively [18]. Shargorodsky et al. reported, in a study with US adolescents, that the OR of high-frequency hearing impairment for the group with a blood lead level over 2 μg/dL was significantly higher compared to a reference group (a blood lead level under 1 μg/dL), that is, 2.22 (95% CI = 1.39–3.56), and that the OR of low-frequency hearing impairment was also significantly higher for the highest quartile group, based on urinary cadmium levels in comparison to the lowest group, that is, 3.08 (95% CI = 1.02–9.25) [17]. Park et al. conducted a study with 448 community-dwelling elderly men in eastern Massachusetts and demonstrated a link between hearing impairment and an increase in the interquartile range of the lead levels in the patella. In their study, the OR was 1.5 (95% CI = 1.1–1.9). In conclusion, evidence that lead and cadmium affect hearing ability within the general population is accumulating steadily [16].

Although previous studies have shown that heavy metals such as lead and cadmium affect hearing ability, the present study, to the best of our knowledge, is the first epidemiologic study conducted on Korean participants to examine the relationship between hearing impairment and cadmium exposure. The ototoxicity mechanism involving cadmium is suggested by only a handful of studies. In a study with rats exposed to water containing cadmium, it was shown that cadmium produces reactive oxygen species in auditory cells and causes loss of mitochondrial membrane depolarization, release of cytochrome c, activations of apoptosis and caspases, and an increase in extracellular signal-regulated kinase activation that ultimately elevates the hearing threshold [13, 14]. A study with US adolescents reported a significant relationship between urinary cadmium and low-frequency hearing impairment. In that study, hearing impairment was defined as an average of hearing thresholds for 0.5, 1, and 2 kHz exceeding 15 dB [17]. In a study with US adults, there was a significant correlation between blood cadmium levels and hearing impairment, with hearing impairment defined as the hearing thresholds for 0.5, 1, 2, and 4 kHz frequencies exceeding 25 dB in one of two ears [18]. Unlike these previous studies, no significant relationship was found between cadmium and hearing impairment in our study, due to several possible reasons. First, this study conducted a different statistical analysis. In the two previously mentioned studies, the data were not categorized by sex, and sex was included as an adjusting variable, unlike the present study. When we performed an additional analysis on the entire data set, not separating it into male and female categories, but including sex as an adjusting variable, we observed that the OR of high-frequency hearing impairment for the highest quartile group with the lowest group as a reference was significant, that is, 1.40 (95% CI = 1.07–1.82). Secondly, the differing results may be due to differences in physical responses caused by variations in ethnicity and lifestyle. Thirdly, the differing results may be due to different definitions and thresholds of hearing impairment used across these studies. Aside from these possibilities, our study and the study with US adolescents had different study participants, that is, adults versus adolescents, and differed in how cadmium was measured, namely in blood versus urinary test levels. More research is needed to study the link between cadmium and hearing impairment in the Korean population.

Regarding the link between lead and hearing impairment, many studies have reported results similar to the current study findings, unlike the case with cadmium. However, while the mechanisms through which lead influences the auditory system are not yet clearly known, chronic lead exposure is known to be toxic to the central and peripheral nervous systems. By using an auditory brainstem response test, a study has shown that lead exposure affects the conduction function of the peripheral nervous system along the auditory pathways [33]. Likewise, Jones et al. reported that lead exposure changes the axonal structure and function of the brainstem auditory nuclei [12]. An alternative hypothesis is that toxic metals affect intracellular calcium homeostasis [34] and accordingly, chronic lead exposure induces auditory hair cell death [35].

The current study findings support the case that even a low level of lead can negatively affect the hearing condition of the Korean general population and that, therefore, an effort should be made to reduce environmental lead exposure. The fourth quartile groups based on blood lead levels demonstrated a risk for poorer hearing in comparison to the first quartile groups. Currently, the Safety Standards of the Occupational Safety and Health Administration (OSHA) specifies 38.6 μg/dL for lead and 5 μg/L for cadmium as blood level thresholds. None of the 6409 participants in the present study exceeded the recommended threshold for lead, and only 6 exceeded it for cadmium. Accordingly, the current study findings suggest that the risk of hearing impairment is present even at a level below the OSHA threshold recommendation for lead. Evidence has been advanced to demonstrate that even where blood lead and cadmium levels are below the respective recommended threshold, they can still lead to chronic kidney disease, peripheral arterial disease, and hypertension [36–38]. Studies conducted in the US have already suggested the possibility of hearing impairment in adolescents and adults following lead and cadmium exposure at a level below their respective recommended thresholds [17, 18].

An advantage of the present study is that it was conducted on a representative sample of the Korean population, and the findings can be generalized to the entire Korean population. Although evidence for the link between hearing impairment and heavy metals, such as lead and cadmium, is accumulating, most studies have been limited in that they were either animal studies or based on a small sample size [12–16]. Epidemiologic studies targeting the general population, such as this study, are rare.

### Limitations

There are several limitations to this study. First, participants’ past work history was not considered. Secondly, the level of noise exposure was not accurately measured and was based on self-reported data, meaning that information bias could have been present. In addition, the participants might have not accurately recalled their noise exposure history over the course of their lives. Thirdly, information pertaining to additional factors that could affect hearing ability, for example, congenital diseases, middle ear diseases, exposures to physical trauma, medication, and toxic substance history, was not included in the original KNHANES dataset. Fourthly, although the study used secondary data that was representative of the Korean population (KNHANES), it was a cross-sectional study. Hence, a causal relationship between hearing impairment and heavy metals could not be clearly identified.

To overcome these limitations in future research, work history, medical history, noise exposure, physical trauma, medication, and toxic substances should be examined and the noise level in the workplace should be measured. Additionally, a cohort study should be conducted that includes people not exposed to occupational and environmental noise.

## Conclusion

In the Korean population, exposure to low-level lead is significantly associated with high-frequency hearing impairment in both men and women. Therefore, lead exposure should be closely monitored to protect citizens’ health.

## Abbreviations

BMI: Body mass index
CI: Confidence interval
KNHANES: Korea National Health and Nutrition Examination Surveys
OR: odds ratio
OSHA: Occupational Safety and Health Administration
WHO: World Health Organization
